# Foliar Morphoanatomical and Phytochemical Variations Shape Resistance to Key Insect Herbivores and Leaf Quality in *Cyclocarya paliurus*

**DOI:** 10.3390/plants14162495

**Published:** 2025-08-11

**Authors:** Zhanhong Xu, Wanxia Yang, Xulan Shang, Xiangxiang Fu, Caowen Sun, Shengzuo Fang

**Affiliations:** 1 State Key Laboratory of Tree Genetics and Breeding, Nanjing Forestry University, Nanjing 210037, China; stephexu@gmail.com (Z.X.); yangwanxia@njfu.edu.cn (W.Y.); scw19871217@126.com (C.S.); 2 Co-Innovation Centre for Sustainable Forestry in Southern China, Nanjing Forestry University, Nanjing 210037, China; shangxulan@njfu.edu.cn (X.S.); xxfu@njfu.edu.cn (X.F.); 3 National Key Laboratory for the Development and Utilization of Forest Food Resources, Nanjing Forestry University, Nanjing 210037, China

**Keywords:** wheel wingnut, leaf trait, secondary metabolite, genotype variation, herbivory damage ratio, comprehensive assessment

## Abstract

To reveal the effects of genotype–herbivore interactions on leaf quality, foliar variations in phytochemicals, morphoanatomy, and herbivory damage ratio were investigated in a *Cyclocarya paliurus* (Batalin) Iljinsk. (Juglandaceae) germplasm resources bank. Results showed less herbivory damage in genotypes with a higher leaf thickness, but more herbivory damage in genotypes with a higher leaf stomatal density. Herbivory damage ratios were significantly correlated with the contents of leaf secondary metabolites, whereas the response of secondary metabolites to insect attack was type-specific and varied between intact leaves and damaged leaves. Based on key indicators of leaf quality (contents of triterpenoids, flavonoids, polyphenols, pterocaryoside A, pterocaryoside B, and cyclocaric acid B), the investigated genotypes were divided into three distinct groups by integrating TOPSIS and cluster analysis, while four genotypes with slight insect damage demonstrated the prioritization for future applications. Our findings lay a foundation for further selection of its superior varieties with both insect resistance and high leaf quality.

## 1. Introduction

It is reported that while growth reductions caused by pests are relatively larger than productivity differences among tree species or genotypes in plantation ecosystems, foresters give this only minor consideration when selecting tree species to plant [1,2]. Some studies indicated that a small loss of leaf tissue could reduce forest productivity by 2–15% on a global scale due to herbivory damage [3]. However, plants have diverse strategies to resist or fight their insect herbivores, including morphological, biochemical, phenological, and physiological variations [4,5,6]. For instance, some species accumulate high levels of chemical compounds to form biochemical defenses through toxicity or physical properties, while other plants seek to reduce herbivore damage via rapid growth and development [7]. Different genotypes in a species adopt various strategies to coexist with insect pests, consequently affecting the partition of resources between growth and defense [8]. Some studies have demonstrated functional trait variations among individual plants within a population [2,9], while only a few studies have been conducted to address the trait variance in a single plant within a species [10,11], which may be closely related to herbivory–plant interactions. There is a growing trend to appreciate that many individual trait variations can represent variations in plant species, such as those occurring among the leaves within a plant; however, our understanding of how these variations relate to herbivory–plant interactions is still limited [12].

Plant secondary metabolites are an important driver of plant–insect community structure among individual plants [2]. However, insect herbivore performance may be influenced by a small variation in leaf chemical traits within a single host plant [4,13]. Plant defenses against insect herbivores can be divided into constitutive defenses and induced defenses [7]. For instance, trembling aspen [*Populus tremuloides* Michx. (Salicaceae)] could produce abundant defense compounds of salicinoid phenolic glycosides and condensed tannins, with salicinoid phenolic glycosides serving as a key defense against lepidopterans [10,14], whereas some secondary metabolites produced in trembling aspen are inducible upon damage [15]. Liao et al. revealed that attacks by major tea pest insects (green leafhopper and tea geometrid) increased the jasmonic acid and salicylic acid contents in tea [*Camellia snensis* (L.) O. Kuntze] (Theaceae) leaves, and modified the tea quality-related non-volatile specialized metabolites (catechins, L-theanine, and caffeine) [16]. Meanwhile, the incidence and preference of insect herbivores are also affected by the foliar morphological traits such as specific leaf area (SLA) and leaf thickness [17]. Ruiz-Carbayo et al. reported that the plants with thick leaves and low SLA generally decrease herbivory levels [18]. It is widely accepted that a plant’s survival in spite of insect damage depends on not only constitutive defensive strategies being employed but also plant-induced resistance; however, plant-induced resistance to insect herbivores is species-specific or genotype-specific in a species [10,19]. Insect herbivory is one of the major factors to impact plant yield and quality, because it is costly to produce and maintain constitutive and induced plant defenses [2,16]. However, information is limited on the effects of insect attacks on plant quality-related traits, especially for plants harvested for their leaves.

Wheel wingnut [*Cyclocarya paliurus* (Batalin) Iljinsk. (Juglandaceae)], a paleoendemic Chinese genus that went extinct in North America and Europe during the Cenozoic period [20], is a multipurpose tree species that is naturally distributed in subtropical areas of China [21]. Owing to the abundant contents of triterpenoids, flavonoids, phenolic acids, and polysaccharides in its leaves, *C. paliurus* was listed as a new food raw material by the National Health and Family Planning Commission of China in 2013. Furthermore, the extract of *C. paliurus* leaves was approved as a new dietary ingredient (NDI) by the Food and Drug Administration of the USA in 2021, and permitted for use in medicines by the Department of Health and Aged Care in the Australian Government in 2023. To meet the market demands for its leaf production, lots of *C. paliurus* plantations have been established in China [22,23]. Over the last 15 years, many studies have focused on superior variety selection and propagation [23,24,25], silvicultural operations (including site selection, planting spacing and pruning, nutrient management and so on) [22,26], and enhancing resistance to abiotic stress in *C. paliurus* [27,28] in order to improve leaf yield and quality in plantations. Unfortunately, the information regarding disease and pest damage on this plant is limited [29], especially since no information is available on the effects of insect attack on its leaf yield and quality.

Based on a three-year investigation in the field germplasm bank of *C. paliurus*, we observed two key insects—*Geisha distinctissima* Walker (Hemiptera: Flatidae) (a piercing–sucking insect) and *Narosa edoensis* Kawada (Lepidoptera: Limacodidae) (a chewing insect)—often attacked its leaves, but the incidences varied among different genotypes (individual trees). Liao et al. reported that insect herbivores not only reduce tea production but also impact the quality-related metabolites [16]. Similarly, *C. paliurus* leaves are also harvested for making health tea as well as for the extraction of pharmaceutical and nutraceutical ingredients [21,23]; however, how insect attack affects the specialized metabolites in its leaves, especially the contents of secondary metabolites, still remains unclear. Based on the investigation of foliar phytochemical and morphoanatomical traits, as well as leaf herbivory damage ratios (involving two key insect herbivores) among the genotypes in the germplasm bank of *C. paliurus*, in this study, we aim to reveal the effects of genotype–herbivore interactions on leaf quality, and investigate the possibility of selecting some superior *C. paliurus* varieties with both resistance to insect herbivores and high leaf quality.

## 2. Results

### 2.1. Variation in Leaf Herbivory Damage Ratio

**Figure 1:**
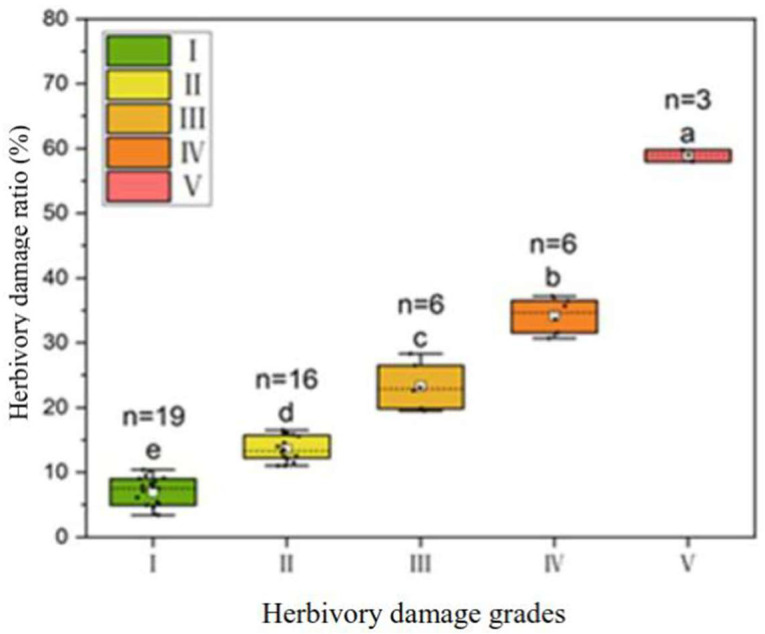
Variation in leaf herbivory damage ratio (HDR) and division of plant herbivory damage grade (HDG) by the k-means clustering. Note: The median is given as short dashed line and the means are given as a white filled square. Different small letters indicate significant differences at *p* < 0.05 among the five HDGs.

The field investigation on the leaf herbivorous damage showed that the herbivory damage ratio (HDR) per plant varied from 2.5% to 59.8% among the 50 genotypes. By means of the k-means clustering, the HDR can be divided into five herbivory damage grades (HDGs), namely grades I, II, III, IV, and V, with the median HDR values of 6.92%, 13.61%, 23.31%, 34.21%, and 58.90%, respectively (Figure 1).

One-way ANOVA indicated there existed a significant difference in the HDR among the five HDGs (*p* < 0.05). Of the 50 individual trees investigated, 38.0% of the trees were slightly damaged by the leaf insect herbivory (grade I), 44.0% of the trees were moderately damaged (including grades II and III), and 18% of the trees were heavily damaged (including grades IV and V), showing a genotype variation in resistance to leaf herbivory damage.

### 2.2. Variations in Leaf Morphoanatomical Characteristics

**Figure 2:**
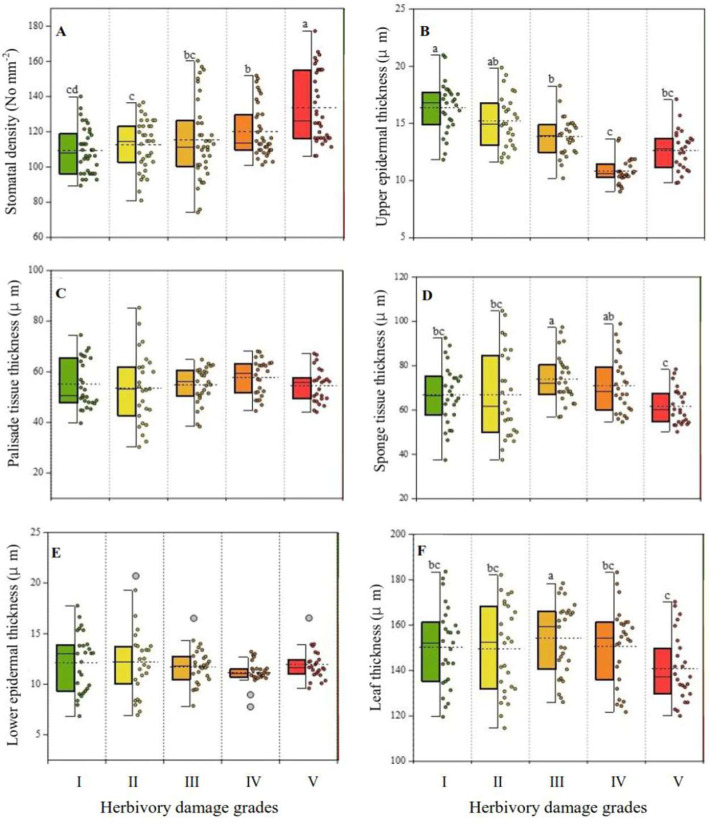
Variations in stomatal density and anatomical structure of intact leaves among the five herbivory damage grades (n = 15). The short-dashed line represents the mean values in each index, while different small letters indicate significant differences at *p* < 0.05 among the five HDGs. (**A**) stomatal density; (**B**) upper epidermal thickness; (**C**) palisade tissue thickness; (**D**) sponge tissue thickness; (**E**) lower epidermal thickness; (**F**) leaf thickness.

One-way ANOVA showed that there were significant differences in stomatal density, sponge tissue thickness, upper epidermal thickness, and leaf thickness among the intact leaves of five HDGs (*p* < 0.05), while no obvious differences were observed in lower epidermal thickness and palisade tissue thickness (*p* > 0.05). As shown in Figure 2A, the average of leaf stomatal density increased with increasing damage grade, but a significant difference was only detected between slightly damaged leaves (grade I) and heavily damaged leaves (including grades IV and V). Compared with grade I, stomatal densities in moderately damaged (including grades II and III) and heavily damaged leaves increased by 3.75% and 15.43%, respectively.

In contrast, the variation in upper epidermal thickness almost showed a decreased trend as the herbivorous damage enlarged (Figure 2B). Compared with grade I, upper epidermal thicknesses in moderately damaged and heavily damaged leaves were reduced by 11.46% and 28.09%, respectively. However, both sponge tissue thickness and leaf thickness showed a similar variation pattern, and the highest values were achieved in moderately damaged leaves, reaching 70.78 μm for sponge tissue thickness (Figure 2D) and 152.79 μm for leaf thickness (Figure 2F), respectively.

### 2.3. Variations in Leaf Secondary Metabolites

**Figure 3:**
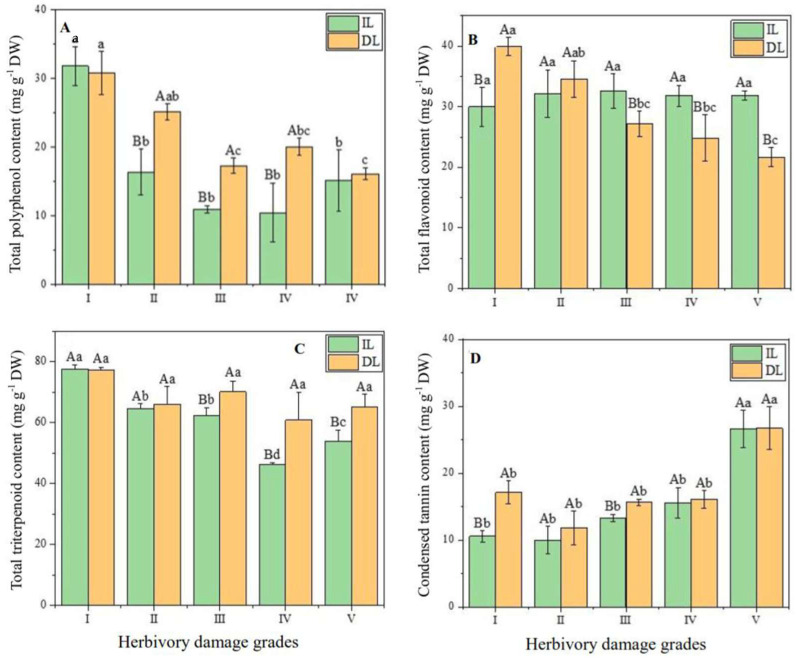
Variations in leaf contents of total polyphenols (**A**), total flavonoids (**B**), total triterpenoids (**C**), and condensed tannins (**D**) among the five herbivory damage grades (HDGs) as well as between intact leaf (IL) and damaged leaf (DL) for each HDG. Different small letters indicate significant differences at *p* < 0.05 among the five HDGs for the same leaf type (i.e., ILs or DLs), while different capital letters indicate significant differences between ILs and DLs for each HDG (*p* < 0.05).

Herbivory damage significantly altered total contents of polyphenols, flavonoids, and triterpenoids, as well as condensed tannin content in the ILs and DLs (*p* < 0.05), except for total flavonoids in ILs and total triterpenoids in DLs (Figure 3). However, the variation patterns in secondary metabolites differed with increasing the damage levels. For instance, total polyphenol contents in both ILs and DLs showed a downward trend (Figure 3A), whereas an upward trend in ILs and DLs was observed for condensed tannin contents (Figure 3D).

The total polyphenol content in leaves of grade I (averaged by the contents in ILs and DLs) was 48.62%, 130.76%, 98.56%, and 82.57% greater than in grades II, III, IV, and V, respectively. The mean values of total flavonoids and triterpenoids in the leaves ranged from 27.17 mg g^−1^ DW to 38.33 mg g^−1^ DW, and 51.82 mg g^−1^ DW to 77.25 mg g^−1^ DW, respectively, but the highest values were both detected in grade I, and the lowest values were observed in grade IV. In contrast, the condensed tannin content in leaves (averaged by the contents in ILs and DLs) in grades I, II, III, and IV decreased by 49.46%, 60.67%, 46.86%, and 44.80%, respectively, when compared with the value in grade V.

It seems that in most cases, the contents of polyphenols, flavonoids, triterpenoids, and condensed tannins in the DLs were higher than those in the ILs for each HDG (Figure 3), with the exception of total polyphenols and condensed tannins in grade I, and total flavonoids in grades III, IV, and V. Although the differences in secondary metabolites varied for each HDG between ILs and DLs, the contents of polyphenols, triterpenoids, and condensed tannins in the DLs (averaged across five HDGs) were 20.20%, 6.09%, and 15.85% higher than in the ILs, respectively. On the contrary, total flavonoid content in the ILs increased by 20.11%, compared with the content in the DLs.

Similarly, monomer contents of quercetin and kaempferol derivatives, and specific triterpenoids in the ILs and DLs were also obviously influenced by damage levels (*p* < 0.05), except for cyclocaric acid in both ILs and DLs, as well as for isoquercitrin, kaempferol-3-O-glucuronide, pterocaryoside A, pterocaryoside B, and arjunolic acid in the DLs (Table S1). However, the diverse variation patterns in the monomer secondary metabolites were observed to increase the damage levels. For example, the highest contents of pterocaryoside A and pterocaryoside B in the ILs were detected in grades II and IV, respectively, but in grades III and II for the DLs (Table S1).

Monomer contents of quercetin and kaempferol derivatives averaged across the seven monomers were 0.273–0.638 mg g^−1^ DW in the ILs, and 0.148–0.390 mg g^−1^ DW in the DLs, respectively, while mean monomer contents across the four measured triterpenoids were 0.182–1.357 mg g^−1^ DW in the ILs, and 0.109–0.562 mg g^−1^ DW in the DLs. However, the highest mean values were observed in grade IV for ILs and in grade II for DLs, respectively, whereas the lowest values all appeared in grade IV.

### 2.4. Variation in Leaf Quality per Plant

Based on the data of 15 individual trees investigated, weighted averages of total triterpenoids, total flavonoids, total polyphenols, pterocaryoside A, pterocaryoside B, and cyclocaric acid B in the leaves were selected as key quality variables. Using the six quality indicators, a comprehensive assessment for leaf quality and prioritization of 15 genotypes was performed by the TOPSIS method. The values of the distance between the ideal point and each alternative (di^+^), the distance between the negative ideal point and each alternative (di^−^), and relative closeness to the ideal point (C_i_), as well as the performance ranking of the 15 selected genotypes, are shown in Table 1.

**Figure 4:**
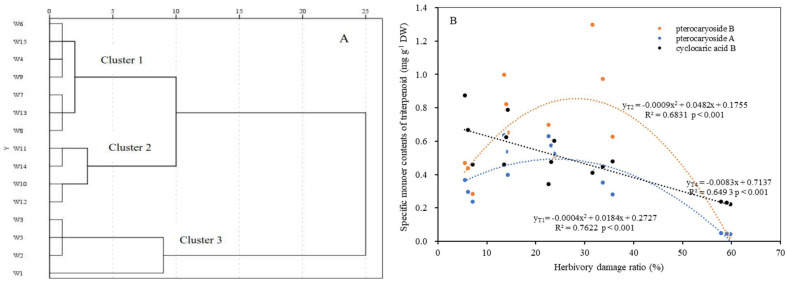
A cluster analysis dendrogram of leaf quality and prioritization based on a comparison of Euclidean distance of relative closeness to the ideal point (C_i_ values) of the 15 genotypes (**A**) and the relationships of the weighted averages of leaf pterocaryoside A (y_T1_), pterocaryoside B (y_T2_), and cyclocaric acid B (y_T4_) contents per to herbivory damage ratio (x) (**B**).

A hierarchical cluster analysis indicated that three distinct groups can be divided for the 15 selected genotypes based on Euclidean distance of C_i_ values (Figure 4A). Cluster 1 consisted of seven genotypes with the values (C_i_) of relative closeness to the ideal point between 0.3343 and 0.495, showing the middle leaf quality. Cluster 2, including four genotypes (W10, W11, W12, and W14), showed poor leaf quality with C_i_ values of 0.028–0.250. Cluster 3 also included four genotypes (W1, W2, W3, and W5) with the C_i_ values of 0.661–0.947, indicating the best leaf quality. Our results suggest that the leaf quality is associated with the insect herbivory attack, whereas four genotypes (individual trees) with slight insect damage (i.e., W1, W2, W3, and W5) demonstrate the prioritization for future asexual reproduction and applications in similar planting areas.

It is worth pointing out that, as important leaf quality indicators in *C. paliurus*, although the weighted averages of pterocaryoside A, pterocaryoside B, and cyclocaric acid B per individual showed a decreasing trend with increasing the HDRs (Figure 4B), the quadratic equations could best describe the relationship between pterocaryosides (including A and B) and HDR (*p* < 0.001), indicating that moderate herbivore damage would favor foliar pterocaryoside accumulations per individual in the field.

### 2.5. Relationships of Herbivory Insect Attack to the Measured Traits

Pearson’s correlation analysis, based on 15 genotypes investigated, indicated that positive correlations between HDR and the contents of total flavonoids (*p* < 0.05) as well as between HDR and condensed tannin (*p* < 0.01) were detected in the ILs, whereas negative relationships were found between HDR and the contents of total triterpenoids (*p* < 0.05) and total polyphenols (*p* > 0.05) (Table 2). However, a significantly negative correlation between HDR and total flavonoid content (*p* < 0.001) as well as between HDR and total polyphenol content (*p* < 0.01) was observed, suggesting that the responses of secondary metabolite accumulation to herbivory insect attacks in the DLs would be different from those in the ILs for the same individual tree.

To reveal the relationship between HDR and secondary metabolite accumulation per tree, the weighted average contents of key secondary metabolites per individual were calculated and correlated with the HDR per individual tree (Table 2). The results showed a negative relationship between HDR and the weighted average of total flavonoid content (*p* > 0.05), total triterpenoids (*p* < 0.05), and total polyphenols (*p* < 0.05), whereas a significantly positive correlation was found between HDR and the weighted average of condensed tannin per tree (*p* < 0.01), indicating leaf condensed tannins, as one kind of polyphenols, were most strongly affected by herbivory insect attacks at an individual tree level.

**Figure 5:**
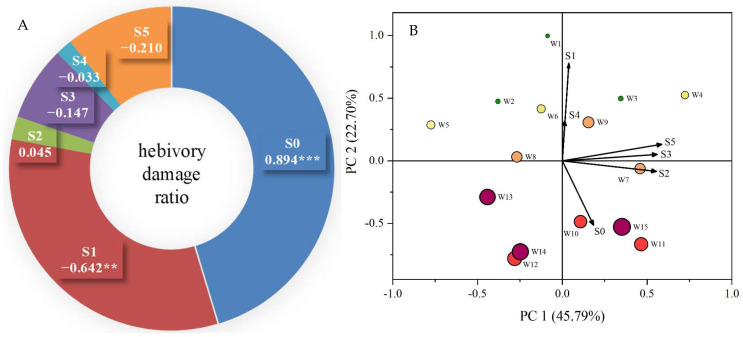
Coefficients between herbivory damage ratios and leaf morphoanatomical characteristics (**A**) and principal component analysis (PCA) of leaf morphological traits in different herbivory damage grades (**B**). S0: leaf stomatal density; S1: upper epidermal thickness; S2: palisade tissue thickness; S3: spongy tissue thickness; S4: lower epidermal thickness; S5: leaf thickness. Filled circles with different colors indicate individuals of different HDGs, with a larger circle area indicating a higher herbivory damage ratio per individual. ** and *** indicate significance at 0.01 and 0.001 levels, respectively.

In the present study, we also evaluated the relationship between the herbivory damage ratio in individual trees (genotype) and the morphoanatomical traits of intact leaves (IL) in the corresponding individuals (Figure 5). Correlation analyses indicated that the HDR was negatively related to upper epidermal thickness (r = −0.642, *p* < 0.01), leaf thickness (r = −0.210, *p* > 0.05), and spongy tissue thickness (r = −0.147, *p* > 0.05), but a significantly positive correlation was detected between HDR and leaf stomatal density (r = 0.894, *p* < 0.001) (Figure 5A).

Moreover, to determine the extent to which morphoanatomical traits affect HDR, additional principal component analysis (PCA) was performed. The first two PCAs, analyzed from six variables (six leaf morphoanatomical traits), explained 68.5% of the total variation (PC1 explained 45.8% and PC2 explained 22.7%, respectively) (Figure 5B). Based on the loading coefficients of the first two principal components (Table S2), the first component (PC1) mainly reflected the traits of leaf thickness, spongy tissue thickness, and palisade tissue thickness, while the second component (PC2) largely mirrored the traits of upper epidermal thickness and leaf stomatal density (when the leaf trait with loading coefficient > 0.5 was included).

## 3. Discussion

### 3.1. Responses of Leaf Secondary Metabolites to Herbivory Insect Attacks

Plants, as sessile organisms, are susceptible to a variety of biotic and abiotic stresses throughout their life cycles, and have evolved multiple defense mechanisms against these tough environmental constraints [5,30,31]. Plant health depends on the natural immunity of the plant that rests with the induction of preformed defense responses, such as morphoanatomical, biochemical, physiological, and genetic variations [31]. However, many studies suggest that secondary metabolites have great potential in providing defense against abiotic as well as biotic stresses [2,32]. These secondary metabolites especially provide defense via toxicity, acting as a feeding deterrent, and so on under biotic stresses [30]. Therefore, plant secondary metabolites not only directly play a key role in the development, protection, and environmental adaptation of plants, but also could be a potential tool to investigate biotic stress tolerance in plants [31].

It was reported that insect herbivores choose more than one host for their nutrition and prefer to select plants with a low concentration of secondary metabolites [5,30,32]. Our results demonstrated that the damage levels significantly altered the total contents of polyphenols, flavonoids, and triterpenoids, as well as condensed tannin content (a kind of non-flavonoid polyphenol) (Figure 3), but showed divergent variation patterns between the two leaf types sampled (ILs and DLs). As HDG increased, the contents of total polyphenols, total triterpenoids, and condensed tannin in the DLs were higher than those in the ILs, in agreement with the results from tea plants where tea leaves infested by tea green leafhoppers (*Empoasca onukii* Matsuda) had greater amounts of internal linalool and emitted linalool than those of intact tea leaves [33]. However, total flavonoid contents showed a different variation pattern (Figure 3B), implying that the defense response to the herbivory attack may vary among the various secondary metabolites, which is confirmed by the variations in monomer contents of flavonoids and triterpenoids between ILs and DLs (Table S1).

Some studies demonstrated that the defense compound accumulation differed not only among plant species, but also among the genotypes within a species, as well as among the crown strata within a genotype [2,10]. However, in most cases, genotypic variations shaped the defense compound accumulation and biotic stress tolerance in plants [9,34], while crown position effects were negligible [10]. Our field investigation showed an obvious variation in HDR among the *C. paliurus* genotypes (Table S1). Plants can produce a large number of secondary metabolites (such as phenolics, flavonoids, saponins, terpenoids, and alkaloids) to serve as defense compounds against herbivores and microorganisms, whereas their functional mechanisms vary among various secondary metabolites as well as among different biotic stresses [31]. For instance, it was reported that phenolic compounds protect plants from biotic stresses with two stages, i.e., the phenols accumulation at the site of infection, and the biosynthesis of specific polyphenolics and other secondary metabolites that restrict the damage at the infection site [32]. Unfortunately, the present field study did not allow us to separate the response of secondary metabolites to each type of herbivory insect attack (piercing–sucking or chewing). Moreover, this field investigation did not permit us to distinguish constitutive from induced chemical defenses. As herbivore feeding behavior and constitutive/inducible chemical compounds of plant tissues are heritable traits [2], further research should be conducted under controlled conditions to reveal the functional mechanisms of different secondary metabolites against specific herbivory insect attacks in *C. paliurus*.

### 3.2. Relationships Between Leaf Morphoanatomical Traits and Insect Damage

To respond to different environmental conditions, variations in leaf morphological traits are often observed among the genotypes of a species as well as among the crown positions within an individual tree [10,17,18]. It was reported that variations in leaf traits (including morphological and chemical traits) can potentially affect the incidence and preference of insect herbivores [10,17,35]. However, most of the morphological studies focused on specific leaf area (SLA), leaf thickness, plant height, leaf dry matter content, and leaf area [6]. For instance, plants generally exhibit conservative strategies, such as thick leaves with low SLA to decrease herbivory levels [18,36]. Unfortunately, less information is available on how leaf anatomical traits potentially influence the incidence and preference of insect herbivores.

Leaf stomata density plays a key role in responding to changing environmental conditions, not only for plants to effectively balance water conservation with efficient photosynthesis but also to influence plant resistance to microbial and insect attacks [37,38]. In the present study, the PCA results showed that the genotype with higher leaf thickness showed a lower herbivory damage ratio, whereas the genotype with higher leaf stomatal density was more susceptible to the insect attacks (Figure 5B). Our results highlight that plants with a higher stomatal density may be more susceptible to certain pests, even if further studies are required to reveal their interaction mechanisms.

### 3.3. Effects of Herbivory Damage Ratio on Leaf Quality per Plant

Plant secondary metabolites, as natural bioactive compounds, are an important source for pharmaceuticals, functional foods, cosmetics, agriculture, and so on due to their health-promoting functions and prevention of some diseases [5,39]. Some recent studies have been conducted to investigate the responses of quality specialized metabolites in tea leaves to major tea insect attacks and revealed the possible mechanisms to promote metabolite formation under insect attack [16,33,40]; however, these studies were conducted under laboratory conditions. In practice, however, plants may be attacked by more than one insect together (including piercing–sucking and chewing herbivores), and how these herbivore damages affect product quality per plant has been less studied.

Many studies have demonstrated that the contents of triterpenoids, flavonoids, phenolics, and polysaccharides, as well as some specific monomer contents of triterpenoids (such as pterocaryosides and cyclocaric acids), are important indicators for assessing the leaf quality of *C. paliurus* [24,26]. However, fewer studies have been conducted to evaluate the effects of herbivory insect attacks on the leaf quality of this species. In the present study, we evaluated the relationship between HDR (largely caused by two insects) and secondary metabolite accumulations in *C. paliurus* leaves at the individual tree level, and the results showed that there existed diverse relationships between the weighted average of various secondary metabolite contents in the leaves and HDR (Table 2 and Figure 4B). For instance, the accumulations of total triterpenoids, total polyphenols, and cyclocaric acid in the leaves per tree would decrease as the HDR increases (Table 2), whereas moderate damage by insect herbivores (including grades II and III) may induce the accumulation of pterocaryoside compounds in *C. paliurus* leaves (Figure 4B).

Insect herbivores are one of the most important factors affecting crop yield quality, although integrated management strategies could minimize the pest population [41]. For instance, insect herbivores lead to 11−55% loss in tea production [16,41], and affect tea quality by modifying the contents of specialized metabolites and their distribution among tissues. Our results indicated that the genotypes with slight attack by insects showed a higher leaf quality (Table 1), indicating that the selection of suitable *C. paliurus* genotypes or cultivars plays a vital role in improving leaf quality in practice.

In accordance with feeding modes, insect herbivores are separated into piercing–sucking and chewing species, which have different impacts on plant performance [16]. Nevertheless, our study was only conducted in field conditions (using a germplasm resource bank of *C. paliurus*). The effects on leaf quality are the combined result of a piercing–sucking herbivore (*G. distinctissima*) and a chewing herbivore (*N. edoensis*), making it hard to isolate the key effects from the two types of herbivory damage. Furthermore, it remains obscure whether various herbivore attacks (such as chewing herbivores and piercing–sucking herbivores) would elicit different responses in inducing accumulations of different secondary metabolites, which in turn affect leaf quality in *C. paliurus*. In any case, our results confirm that there are intraspecific variations in functional leaf traits among individual trees of *C. paliurus*, which can impact the damage ratios of associated insect herbivores as well as its leaf quality. The findings from this study not only deepen our understanding of how foliar phytochemical and morphoanatomical variations shape resistance to key insect herbivores and leaf quality in *C. paliurus* but also highlight the application potential in selecting superior varieties with both insect resistance and high leaf quality.

## 4. Materials and Methods

### 4.1. Study Site and Plant Materials

We conducted the research in a *C. paliurus* germplasm resources bank located at the Baima Teaching Experimental Base of Nanjing Forestry University (119°09′ E, 31°35′ N). The site has a subtropical monsoon climate with an annual average precipitation and average temperature of 1087.4 mm and 15.4 °C, respectively. The soil is yellow-brown with a pH value of 5.69 and a bulk density of 1.30 g cm^−3^ at a soil depth of 40 cm. The *C. paliurus* germplasm resources have been collected from throughout its natural distribution areas since 2001, propagated from the seeds or by grafting, and then planted with 2.0 × 3.0 m spacing in a completely randomized block design. Until now, 36 provenances, 240 families, and 19 clones of *C. paliurus* have been established in the germplasm resources bank [23].

In this study, a total of 358 individual trees that originated from 63 families were used to conduct a preliminary investigation. The seeds of the 63 families were all collected in October 2014 from 21 provenances, and the seedlings for each family were planted on the germplasm resources bank in the Spring of 2017. Based on the preliminary assessment of 358 individual trees, only 50 superior trees with selective breeding potential were selected for detailed investigation of insect herbivore damage, and then 3 genotypes (i.e., 3 individual trees) for each herbivore damage grade (Table S3) were chosen for detailed analysis of leaf secondary metabolite contents and morphoanatomical variations.

### 4.2. Field Investigation on Insect Herbivores and Leaf Damage

**Figure 6:**
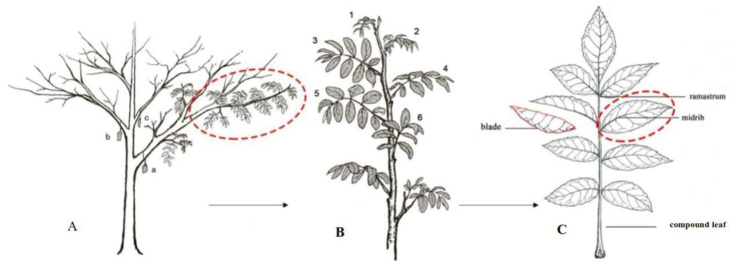
Schematic diagram of branch positioning and leaf sampling for herbivory damage grade assessment and subsequent leaf sampling for analysis. (**A**) a, b and c represent selected branches for sampling; (**B**) 1, 2, 3, 4, 5 and 6 show the order of compound leaves on a sampled branch (in red circle); (**C**) a compound leaf for leaflet sampling.

Over the 3-year observation period from 2019 to 2021 in the germplasm resources bank, we found the key insect herbivores attacking *C. paliurus* leaves are *G. distinctissima* (a piercing–sucking herbivore) and *N. edoensis* (a chewing herbivore), which mainly occur during the period from mid-June to end-July (Table S4). To assess the variation in herbivory damage grade and subsequent leaf sampling for further analysis, three primary lateral branches were randomly selected from the low and middle parts of the tree crown for each selected tree (genotype), and marked as a, b, and c in early June 2021 (Figure 6A). Then, 300–500 leaflets were randomly collected from the three marked branches for each tree on July 24, 2021. Leaflets with incomplete and irregular or spiky and faded spots were defined as damaged leaflets (DLs), and undamaged leaflets were defined as intact leaflets (ILs). Finally, the leaf herbivory damage ratio (HDR) was calculated for each selected individual tree, according to the following equation:

HDR (%) = [NDL_a+b+c_/(NDL_a+b+c_ + NIL_a+b+c_)] × 100%

where NDL_a+b+c_ indicates the total number of DLs observed in the leaves sampled from the marked branches, while NIL_a+b+c_ indicates the total number of ILs observed.

### 4.3. Leaf Sampling Method

After the HDR assessment for each genotype, three compound leaves—labeled 4, 5, and 6—in Figure 6B were collected from three marked lateral branches (a, b, and c), and a total of 15 trees (genotypes) were sampled (i.e., three genotypes close to median HDR value were selected for each damage grade). The fifth leaflet without damage on the collected compound leaves was used to measure the morphoanatomical characteristics (Figure 6C), whereas the other leaflets on the sampled compound leaves, identified as DLs and ILs, were collected for determining the contents of secondary metabolites, respectively.

### 4.4. Measurements of Leaf Morphoanatomical Characteristics

A nail polish imprint method was adopted to measure stomatal characteristics of the sampled leaflets [42]. Briefly, about 1 cm^2^ area was selected from the abaxial surface of the leaf (avoiding midrib and large veins), and evenly applied a thin layer of transparent nail polish. After natural drying, the imprinted film was peeled off, mounted on a glass slide, and observed by a Biological Microscope BX53 (Olympus, Tokyo, Japan). Stomatal counts were performed in 3 randomly chosen microscopic fields for each leaflet sample, and then the number of stomata per field was converted to the number of stomata mm^−2^, namely stomatal density.

For anatomical observations, a small leaf section (0.4 cm × 0.6 cm, avoiding the main vein and edge of the leaflets) was cut from the same leaflet used to measure stomatal characteristics, and the conventional paraffin section method was adopted to make sections [43]. After fixing them with FAA fixative, dehydrating with alcohol of different gradients, xylene transparent, and embedding them in paraffin, the 4 µm thick sections were observed under a Biological Microscope BX53 (Olympus, Tokyo, Japan). Finally, the upper epidermis thickness (UET, μm), lower epidermis thickness (LET, μm), palisade tissue thickness (PTT, μm), spongy tissue thickness (STT, μm), and leaf thickness (LT, μm) were obtained based on the corresponding analysis software. All measured parameters for each leaf damage grade included three biological replicates (three individual trees) with 27 observations, and a total of 135 observations were made for five leaf damage grades.

### 4.5. Extraction and Analysis of Leaf Secondary Metabolites

To determine the contents of leaf secondary metabolites, the sampled leaves (0.8 g, including ILs and DLs) were extracted with 10 mL of 70% ethanol for 1 h at 70 °C, followed by ultrasonic extraction for 45 min [44]. The contents of total polyphenol (expressed as the gallic acid equivalent in mg g^−1^ dry sample) were measured as described by Alothman et al. [45], while total flavonoids (expressed as the rutin equivalent in mg g^−1^ dry sample) were determined according to the method described by Zhou et al. [46]. The total triterpenoid content was determined using the colorimetric method and expressed as the oleanolic acid equivalent in mg g^−1^ dry sample [47,48].

After centrifugation at 10,000 rpm for 10 min, all extractions were filtered through a 0.22 µm polytetrafluoroethylene (PTFE) filter prior to high-performance liquid chromatography analysis (HPLC). The monomer (individual) contents of flavonoids and triterpenoids in the sampled leaves were detected by the Agilent 1200 series HPLC system (Santa Clara, CA, USA) based on the procedures described by Cao et al. (2017) [44]. The reference standards of isoquercitrin, kaempferol-3-O-glucuronide, quercetin-3-O-glucuronide, quercetin-3-O-rhamnoside, quercetin-3-O-galactoside, kaempferol-3-O-glucoside, and arjunolic acid (purity > 98%) were purchased from BioBioPha Co., Ltd. (Kunming, China), whereas cyclocaric acid B, kaempferol-3-O-rhamnoside, and pterocaryoside A and pterocaryoside B (purity > 98%) were isolated and purified from the laboratory of China Pharmaceutical University (Nanjing, China). The HPLC chromatograms of the representative sample solution and the responding standard solution containing the flavonoid and triterpenoid compounds are shown in Figure S1.

Condensed tannin contents in the leaves were determined by the vanillin method with minor modification [49]. Briefly, 50 mg of freeze-dried powder of the samples, stored under nitrogen at −20 °C, was accurately weighed, put into a stoppered test tube, and then, 5 mL of 70% methanol was added to the tube. After shaking well, leaving undisturbed at room temperature for 24 h, and centrifuging at 5000 r min^−1^ for 10 min at 4 °C, the supernatant was taken as the extraction solution for further analysis. The absorbance was read at 510 nm, and the content of condensed tannin was expressed as the catechin equivalent in mg g^−1^ dry sample.

In terms of secondary metabolite contents measured in damaged leaflets (DL) and intact leaflets (IL) mentioned above, the weighted average of each leaf’s secondary metabolite content per individual tree (WAC) was calculated according to the following equation:

WAC = [C_DL_ × HDR + C_IL_ × (100 − HDR]/100

where C_DL_ and C_IL_ represent each secondary metabolite content in the damaged leaflets and intact leaflets, respectively, while the HDR is the leaf herbivory damage ratio (%) for each genotype (individual tree).

### 4.6. Comprehensive Assessment of Leaf Quality

Using the weighted average of leaf secondary metabolite contents selected in each genotype as variables, the Technique for Order of Preference by Similarity to Ideal Solution (TOPSIS) [50], a multi-criteria assessment method based on relative closeness to the ideal point (C_i_), was used to assess the effects of herbivory damage ratio on leaf quality of *C. paliurus* in this study. The division of every option from the positive and negative perfect arrangement (di^+^, di^−^), as well as the ideal point (C_i_), was standardized and calculated using the equations described by Fang et al. [51].

### 4.7. Statistical Analyses

SPSS 20.0 version (SPSS, Inc., Chicago, IL, USA) was employed for statistical data analysis, while Origin software (OriginLab, OriginPro 2021, USA) was used for creating figures. The k-means clustering was adopted to divide the leaf herbivory damage grades for each individual tree, and one-way ANOVA followed by Duncan’s test was performed to compare the differences in the measured indexes among the different damage grades at *p* < 0.05. However, differences between the ILs and the DLs within each leaf damage grade were compared using Student’s *t* test. To obtain the magnitude of each indicator’s contribution and eigenvectors, a principal component analysis (PCA) was performed. All data were presented as mean ± standard deviation.

## 5. Conclusions

On the basis of the HDR investigation of 50 genotypes in the *C. paliurus* germplasm resources bank, 15 genotypes under different HDGs were selected to investigate foliar variations in secondary metabolites and morphoanatomical traits, as well as the leaf quality difference. Correlation analysis and PCA indicated that the genotypes with higher leaf thickness showed a lower herbivory damage ratio, whereas the genotypes with higher leaf stomatal density were more susceptible to the herbivory attacks. Herbivory attacks significantly shaped foliar contents of polyphenols, flavonoids, triterpenoids, and condensed tannins, as well as monomer contents of quercetin and kaempferol derivatives and specific triterpenoids, but the response of secondary metabolite accumulation to herbivory attack varied for each secondary metabolite and between intact leaves and damaged leaves. Based on six key indicators of leaf quality, a comprehensive assessment was made for 15 genotypes, and the genotypes with low HDRs (such as W1, W2, W3, and W5) displayed higher leaf quality. Our findings provide new references for further selection of superior varieties with high leaf quality as well as insect resistance in *C. paliurus*.

## Figures and Tables

**Table 1:** A comprehensive assessment for leaf quality and prioritization of 15 selected genotypes of *C. paliurus* by the TOPSIS method.

Individual Code	Herbivory Damage Ratio (%)	d_i_^+^	d_i_^−^	C_i_	Ranking Order
W1	7.1	2.089	37.407	0.947	1
W2	6.1	12.967	31.529	0.709	2
W3	5.5	13.801	28.855	0.676	3
W4	14.3	21.080	17.076	0.448	7
W5	14.0	13.239	25.869	0.661	4
W6	13.5	19.372	18.992	0.495	5
W7	23.8	26.985	13.708	0.337	11
W8	23.1	26.495	14.792	0.358	9
W9	22.7	23.384	17.054	0.422	8
W10	35.7	37.478	1.081	0.028	15
W11	33.7	29.080	9.718	0.250	12
W12	31.5	33.856	4.530	0.118	14
W13	59.8	27.373	14.304	0.343	10
W14	59.0	30.810	7.722	0.200	13
W15	57.9	19.530	19.064	0.494	6

Note: W1–15 represent different genotypes (individual trees); di^+^, di^−^, and C_i_ are the distance between the ideal point and each alternative, the distance between the negative ideal point and each alternative, and relative closeness to the ideal point, respectively.

**Table 2:** Pearson’s correlation coefficients between herbivory damage ratios and the total contents of secondary metabolites in various leaf types (n = 15).

Index	Secondary Metabolites				
	Leaf Types	Total Flavonoids	Total Triterpenoids	Total Polyphenols	Condensed Tannin
Herbivory damage ratio	Intact leaf	0.498 *	−0.571 *	−0.265	0.722 **
	Damaged leaf	−0.822 ***	−0.536 *	−0.721 **	0.540 *
	Weighted average	−0.370	−0.584 *	−0.581 *	0.681 **

Note: *, **, and *** indicate significance at 0.05, 0.01, and 0.001 levels, respectively.

## Data Availability

Data will be made available on request.

## Supplementary Materials

The following supporting information can be downloaded at: https://www.mdpi.com/article/10.3390/plants14162495/s1, Figure S1: HPLC chromatograms of the representative sample solution (top) and a mixed standard solution containing the 16 quantitative compounds (bottom); Table S1: Variations in monomer contents of flavonoids and triterpenoids in the sampled leaves among the five herbivory damage grades; Table S2: Loading coefficients of the first two principal components; Table S3: Basic information of selected 15 genotypes of *Cyclocarya paliurus* for leaf phytochemical and morphoanatomical analysis; Table S4: The two types of damage symptoms and corresponding insect herbivores in the *Cyclocarya paliurus* germplasm resources bank.

## Abbreviations

The following abbreviations are used in this manuscript:

HDR	Herbivory damage ratio
HDG	Herbivory damage grade
ILs	Intact leaflets
DLs	Damaged leaflets
SLA	Specific leaf area
TOPSIS	Technique for Order of Preference by Similarity to Ideal Solution
