# Supplementary material for: Foliar Morphoanatomical and Phytochemical Variations Shape Resistance to Key Insect Herbivores and Leaf Quality in Cyclocarya paliurus
Source: Plants (Basel). 2025 Aug 11;14(16):2495. doi: 10.3390/plants14162495 (PMC12389510; doi:10.3390/plants14162495)
Supplement: Supplementary file 1 [file plants-14-02495-s001.zip › plants-3798651-supplementary.pdf]

Supplementary Materials:

Foliar morphoanatomical and phytochemical variations shape the resistance to key insect herbivores and leaf quality in *Cyclocarya paliurus*

Zhanhong Xu[1,†], Wanxia Yang[1,3,†], Xulan Shang[2,3], Xiangxiang Fu[2,3], Sun Caowen[1,3], Shengzuo Fang[1,3*]

[a] State Key Laboratory of Tree Genetics and Breeding, Nanjing Forestry University, Nanjing 210037, China

[b] National Key Laboratory for the Development and Utilization of Forest Food Resources, Nanjing 210037, China

[c] Co-Innovation Centre for Sustainable Forestry in Southern China, Nanjing 210037, China

[†] These authors contributed equally to this work.

* Corresponding author:

Dr. Shengzuo Fang

Email: fangsz@njfu.edu.cn

Supplementary Tables:

Table S1. Variations in monomer contents of flavonoids and triterpenoids in the sampled leaves among the five herbivory damage grades

Monomer types		Herbivory damage grades (HDG)									
		I		II		III		IV		V	
		IL	DL	IL	DL	IL	DL	IL	DL	IL	DL
Quercetin and kaempferol derivatives (mg g^{-1} DW)	F1	0.444±0.188AB	0.689±0.197A	1.123±0.081A	0.619±0.125AB	0.304±0.041B	0.308±0.045BC	0.833±0.366AB	0.061±0.033C	0.481±0.256ABB	0.044±0.022C
	F2	0.214±0.114A	0.187±0.127A	0.116±0.014AB	0.105±0.020AB	0.041±0.003B	0.048±0.006B	0.229±0.097A	0.096±0.064AB	0.100±0.004A	0.129±0.055AB
	F3	0.117±0.001B	0.084±0.041AB	0.130±0.012B	0.094±0.025A	0.291±0.055A	0.068±0.006AB	0.150±0.030AB	0.061±0.026AB	0.089±0.015B	0.045±0.004B
	F4	0.079±0.035A	0.056±0.037	0.053±0.004B	0.047±0.004	0.206±0.061A	0.043±0.016	0.169±0.083AB	0.088±0.063	0.094±0.025AB	nd
	F5	0.512±0.355B	0.637±0.149	1.253±0.240A	0.854±0.271	0.145±0.033B	0.521±0.187	1.354±0.212A	0.528±0.181	0.537±0.056B	0.268±0.053
	F6	0.113±0.040B	0.079±0.005B	0.101±0.006B	0.089±0.008B	0.196±0.069B	0.094±0.013B	0.421±0.095A	0.185±0.031A	0.163±0.046B	0.096±0.010B
	F7	1.266±0.475AB	0.674±0.134AB	1.516±0.272A	0.919±0.278A	0.725±0.130B	0.711±0.159AB	1.310±0.237AB	0.515±0.265B	1.262±0.077AB	0.451±0.096B
	**means**	**0.392**	**0.344**	**0.613**	**0.390**	**0.273**	**0.255**	**0.638**	**0.219**	**0.389**	**0.148**
Specific triterpenoids (mg g^{-1} DW)	T1	0.929±0.489AB	0.257±0.198	1.329±0.103A	0.394±0.202	0.808±0.116AB	0.506±0.195	0.432±0.045B	0.312±0.121	nd	0.110±0.057
	T2	1.093±0.453AB	0.398±0.184	1.369±0.289AB	0.734±0.551	0.875±0.065AB	0.524±0.141	2.054±0.716A	0.427±0.326	0.038±0.038B	0.055±0.055
	T3	0.500±0.447B	0.243±0.243	1.472±0.410AB	0.466±0.369	0.473±0.141B	0.246±0.218	2.346±0.402A	0.234±0.158	0.488±0.035B	nd
	T4	0.666±0.342	0.669±0.287	0.441±0.050	0.655±0.231	0.389±0.100	0.499±0.234	0.597±0.016	0.371±0.072	0.202±0.015	0.271±0.002
	**means**	**0.797**	**0.392**	**1.220**	**0.562**	**0.654**	**0.444**	**1.357**	**0.336**	**0.182**	**0.109**

Note: IL: intact leaf; DL: damaged leaf; F1: quercetin-3-O-glucuronide; F2: quercetin-3-O-galactoside; F3: quercetin-3-O-rhamnoside; F4: isoquercitrin; F5: kaempferol-3-O-glucuronide; F6: kaempferol-3-O-glucoside; F7: kaempferol-3-O-rhamnoside; T1: pterocaryoside A; T2: pterocaryoside B; T3: arjunolic acid; T4: cyclocaric acid B. Different capital letters in the same line indicate significant differences among the five HDGs for the IL or DL respectively ($p < 0.05$). nd means not detected.

Table S2. Loading coefficients of the first two principal components

Leaf morphoanatomical traits	Principal component loading coefficients	
	PC1	PC2
leaf thickness (S5)	0.972	0.155
spongy tissue thickness (S3)	0.927	0.060
palisade tissue thickness (S2)	0.920	-0.099
upper epidermal thickness (S1)	0.064	0.910
lower epidermal thickness (S4)	0.024	0.373
leaf stomatal density (S0)	0.305	-0.598

Table S3. Basic information of selected 15 genotypes of *Cyclocarya paliurus* for leaf phytochemical and morphoanatomical analyiss

Code of selected genotypes	Individual origin			Tree growth at age 5		Herbivory damage ratio (%)	Herbivory damage grade
	Provenance (Latitude/Longitude)	Family	Individual	Height (m)	Base diameter (cm)		
W1	Anhui Shitai (29.57 N°/117.56 E°)	Shi	Shi-9	4.7	9.0	7.10	I
W2	Guangxi Guilin (25.62 N°/109.89 E°)	Gui	Gui-26	4.6	10.2	6.10	
W3	Guangxi Guilin (25.62 N°/109.89 E°)	Gui	Gui-8	4.2	8.0	5.50	
W4	Anhui Shitai (29.57 N°/109.89 E°)	Shi	Shi-12	4.9	7.7	14.30	II
W5	Anhui Jinde (30.23 N°/118.45 E°)	Jin	Jin-2	4.1	8.7	14.00	
W6	Zhejiang Fenghuangshan (29.91 N°/121.19 E°)	Feng	Feng-2	3.9	7.7	13.50	
W7	Zhejiang Fenghuangshan (29.91 N°/121.19 E°)	Feng	Feng-1	3.8	6.7	23.80	III
W8	Sichuan Muchuan (28.96 N°/103.9 E°)	Mu	Mu-24	4.2	9.4	23.10	
W9	Hubei Wufeng (30.20 N°/110.67 E°)	Wu	Wu-15	5.0	11.3	22.65	
W10	Hunan Yongshun (29.02 N°/109.85 E°)	Yong	Yong-2	4.1	8.4	35.67	IV
W11	Hunan Yongshun (29.02 N°/109.85 E°)	Yong	Yong-3	3.5	6.5	33.65	
W12	Anhui Jinde (30.23 N°/118.45 E°)	Jin	Jin-1	4.7	8.4	31.53	
W13	Sichuan Muchuan (28.96 N°/103.9 E°)	Mu	Mu-24	4.2	9.4	59.78	V
W14	Sichuan Muchuan (28.96 N°/103.9 E°)	Mu	Mu-6	4.8	10.1	58.96	
W15	Hubei Wufeng (30.20 N°/110.67E°)	Wu	Wu-1	3.6	8.4	57.90	

Table S4. The two type damage symptoms and corresponding insect herbivores in the *Cyclocarya paliurus* germplasm resources bank

Insect species	Damages symptom	Damage period	Damage part
Geisha distinctissima	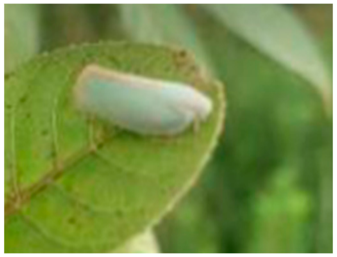	The leaves are damaged by *G. distinctissima* both at the nymph stage between June and July, as well as at the adult stage between July and August each year.	Mainly leaves of wheel wingnut
Narosa edoensis	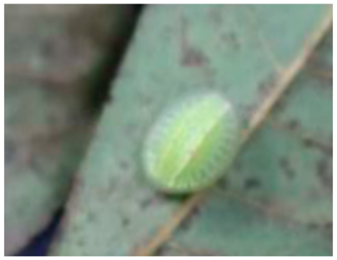	The leaves are mainly damaged by *N. edoensis* at the larval stage between July and August each year.	Mainly mature leaves of wheel wingnut

Note: (upper) The leaf symptom damaged by *Geisha distinctissima* and the corresponding insect at the adult stage (taken on July 24, 2021). (bottom) The leaf symptom damaged by *Narosa edoensis* and the corresponding insect at the larva stage (taken on July 24, 2021).

__Supplementary Figures:__

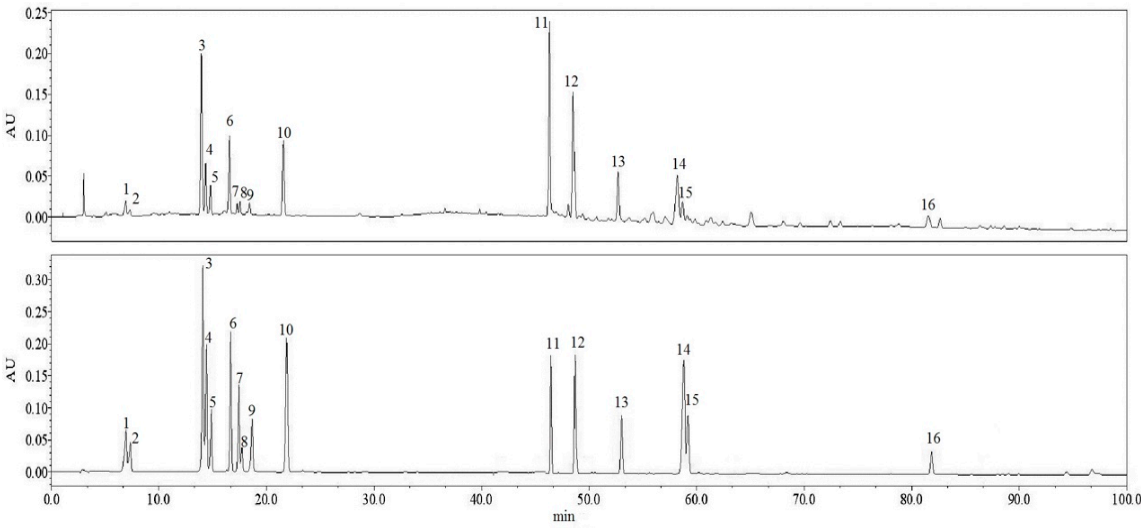

Figure S1. HPLC chromatograms of the representative sample solution (**top**) and a mixed standard solution containing the 16 quantitative compounds (**bottom**). 1: 3-*O*-caffeoyluinic acid; 2: 4-*O*-caffeoyluinic acid; 3: quercetin-3-*O*-glucuronide; 4: quercetin-3-*O*-galactoside; 5: isoquercitrin; 6: kaempferol-3-*O*-glucuronide; 7: kaempferol-3-*O*-glucoside; 8: quercetin-3-*O*-rhamnoside; 9: 4,5-di-*O*-caffeoyluinic acid; 10: kaempferol-3-*O*-rhamnoside; 11: arjunolic acid; 12: cyclocaric acid B; 13: pterocaryoside B; 14: pterocaryoside A; 15: hederagenin; 16: oleanolic acid.
